# Handbuch Der Speciallen Pathalogie Und Therapie

**Published:** 1858-05

**Authors:** 


ART. II.—*Handbuch der Speciallen Pathologie und Therapie.* Bearbeitet von Dr. BAMBERGER, in Vien.; Prof. CHIARI, in Prag.; Dr. FALCK, in Marburg; Prof. GRIESINGER, in Stuttgart; Prof. HASSE, in Heidelberg; Prof. HEBRA, in Vien.; Prof. HEUSINGER, in Marburg; Prof. LEBERT, in Zurich; Prof. PITHA, in Prag.; Dr. SIMON, in Hamburg; Dr. STIEBEL, in Frankfurt a M.; Dr. TRAUBE, in Berlin; Prof. R. VIRCHOW, in Wurzburg; Prof. J. VOGEL, in Giessen; Prof. WINTRICH, in

Erlangen. Redigirt von RUD. VIRCHOW, Prof. der Medicin in Wurzburg. Erlangen, 1854. Erste Bande.

*A Manual of Special Pathology and Therapeutics;* Contributed by Dr. BAMBERGER, of Vienna, and a number of Professors and Doctors. Edited by Professor VIRCHOW. Erlangen, 1854. Volume First.

WERE it our intention to pass in review the contents of this handbook, so called, but which, when completed, may be more properly spoken of as an arm-full of volumes—a Library of Practical Medicine, we should apprise our readers that the sixth, and, as we are led to believe, the last volume, is now on our table. This, in common cases, might be supposed to imply that the work had reached its completion; whereas, in fact, the second and third volumes have yet to be written. Perhaps they have been issued since the date of the sixth, which is of 1855, but as yet they have not reached us. Even taking the work as we find it—incomplete, there is abundant material from which, did time and space permit, we would gladly draw for the edification of our readers. The arrangement of subjects and the method of handling them exhibit a certain degree of freshness, if not of originality, which, though it might not be at once appreciated by the American mind, could scarcely fail to command attention and furnish lessons for profitable imitation.

In our subsequent remarks we shall, for the most part, confine ourselves not only to the first volume of this Manual of Pathology and Special Therapeutics, but, also, to one chapter in it, viz. that on "*Plug-formations and Obturations in the Blood-vessels;*" or, as more technically expressed for the professional ear, *Thrombosis and Embolia,* which will include obstruction and obturation. The subject for a long time, even after it had attracted some notice, was left as a field for conjecture, in which scattered facts were imperfectly seen and still more imperfectly understood: sometimes they were framed into a hypothesis; sometimes regarded, and this was long the opinion of the majority, as anomalous, and the accidental accompaniment, or the immediate effect, of the death-agony. This was the case in a more particular manner with the coagula and clots, more or less changed and organized, which were found in the cavities of the heart. But now, thanks to a more careful and minute study of pathological anatomy, and the observations made in this direction by eminent pathologists, both on the continent of Europe and in England, the whole subject is brought before us with a certain degree of distinctness, and an approach to method, which promise, ere long, to furnish materials for the diagnosis of dangerous morbid states of the organism, and sudden and fatal seizures, that had been previously attributed to wrong causes, or were thought not to be susceptible of explanation.

In our endeavors to justify this favorable view, and to show the practical bearing of the inquiry—for practical, nowadays, is the password, no matter what amount of crude and hasty observation and empiricism it allows to circulate—we shall follow, for the most part, the lead of Professor Virchow, the editor and one of the contributors to the "*Handbuch*," and who is also author of the chapter on which we propose to dwell. The series of essays on the morbid changes of the blood, by Virchow, published in 1856, in Frankfurt, show the progressive steps by which he was led to the establishing what might almost be called a body of doctrine on thrombosis and embolia, and of which he has given a summary in the Handbook of Special Pathology and Therapeutics. Many interesting details—experiments and clinical observations—are recorded in the former of these volumes of essays, which are not introduced into the chapter in the handbook; and, as we are not in possession of the former, we shall be found occasionally turning them to account by the aid of the critical labors of one of our transatlantic contemporaries.*

Virchow's readers are better prepared to understand his account of plug-formations and obstructions in the blood-vessels, by previous chapters on Passive and Active Congestion or Fluxion. He prefaces our immediate theme by a careful bibliography from Bonetus to Tuffnell, in the *Dublin Quarterly Journal*, 1853. We must confess to a feeling of disappointment at not finding, among these authorities, the name of Dr. C. D. Meigs, who was the first in the United States to direct attention to this important matter, through the pages of the *Medical Examiner* for 1849, and of his work on Obstetrics; as, also, in his lectures in the Jefferson Medical College. In a brief historical notice, Virchow tells us that, although the fact of the stagnation and coagulation of the blood in the veins had been long noticed, yet that the attention of medical men seems to have been first formally directed to the occurrence by a physician of Nuremberg, named Walter Coiter, (1572,) and next by Marcus Aurelius Severinus, (1616.) Soon, notwithstanding some objectors, as, for example, Kerkring, it became the received belief that the clots which are so frequently found in the heart and blood-vessels of the dead subject had been, beyond doubt, previously coagulated during life. Thus arose that noted doctrine which took for its stand-point polypi of the heart, and which, in all succeeding times, should be received as a warning example against the hasty application of anatomical facts. Asthma, palpitations, and inflammatory affections of the chest were deemed to be inevitable results of the polypi, which were readily carried far into the vessels. At length, after a long duration of this creed, a reaction, which is very appropriately designated by the author

* British and Foreign Medico-Chirurgical Review, July, 1857.

as empirical, took place, and opinions went to the other extreme; so that, in the latter half of the last century, both the Pastas, Andrew and Joseph, spoke always of the blood-clots found in the dead body as a cadaveric phenomenon. It mattered little that Haller and Morgagni, while adverting to the frequent occurrence of cadaveric coagula, spoke, also, of their not seldom occurring through vital processes; and although the example of Hahnemann shows that it is still possible to fall into the error of the sixteenth century even in our own day, yet there prevailed, for a long time, a general prejudice against the possibility of the formation of coagula in the circulating blood of the living body.

This prejudice derived support from the development of the doctrine of the inflammation of the lining membrane of the heart and blood-vessels. After John Hunter had first explained the arterio-phlebitis, such large additions were made to our knowledge on this subject, that Cruveilhier advanced the bold axiom, that phlebitis governs, or is the key to all pathology. Assuredly pathology was a gainer; but the question began to be asked, how it should happen that inflammation would cause coagulation of the blood,—for Cruveilhier declared that inflammation in general is nothing more than a coagulation of blood in the smaller veins, (*capillar-phlebitis.*) With this view was associated the gradually increasing belief in the exudation-doctrine of inflammation; and it was then said that the obstructing coagulation in the vessel was sometimes an exudation alone, and sometimes a mixture of exudation and blood, as had been in part shown by Cruveilhier. As the belief in the coagulating influence of the exudations from the sides of the vessels on the blood became accredited, there followed what in the preceding century was the received hypothesis of heart polypi, viz. a dyscrasia; an acrid *coagulatorium;* a coagulating principle in the blood; and in some cases a spontaneous formation, in others an exudative inflammation. The starting-point of the inquiry was to determine precisely whence was derived this substance, which must possess, to a certain extent, chemical properties, or to ascertain the peculiar change in the composition of the blood on which the coagulation depends. Thus, we find Piorry extending into a wide generalization the view of Cruveilhier on inflammation of the blood, (*hœmitis,*) which Rokitansky adopted, but with many restrictions; while Paget suggests uramia (urine mixture with the blood) as a cause, at least, of the plug-formations of the pulmonary arteries; and, as had been previously done by Leake and Bouillaud, Engel advanced the opinion of the older Greek physicians, of a fermentative principle in the blood.

While the leading opinions were divided between primary inflammation of the circulatory apparatus, (endocarditis, arteritis, phlebitis,) and primary dyscrasia, a third, the mechanical theory, found supporters in Laennec, Legroux, Bouchut, John Davy, and Gulliver; and it was received by Rokitansky as a probable condition. An admission of the mechanical theory of blood-clot in the blood-vessels does not exclude the question of the composition of the blood, or of a change in the vessel. The formation of a coagulum presupposes a change in the circulation, that is, a mechanical change; and the clot will be formed with more or less ease, according to the series of momenta to which the circulation is subjected. It is clear that the clot is due to the presence of fibrine, and that the former will be less frequent and more imperfect in blood poor in fibrine than when this fluid abounds in fibrine. "I can admit," says the author, "a difference in the dissolved material, through the condensation of which arise the coagulated particles of fibrine; and I can also suppose a greater or less predisposition in the blood crasis to form coagula. We would go further," he adds, "and say, that a change in the composition of the blood, as well as a change in the coats of the vessels, implies an alteration in the molecular attraction between the blood and its containing vessels; and that in this state the crasis, either with or without a change in the vessel, may act as well chemically as mechanically, in the production of the coagulum."

Virchow, after this historical review of opinions, enters directly on his immediate subject, by telling us that the coagulation of the blood within the vessels follows the same laws as when it occurs out of them, while the fluid fibrine will be altered in its mode of aggregation and become solid without the access of atmospherical air. It is converted shortly into a full, soft, and red clot, loosely connected with the coat of the vessel, and which, at the most, may seem to be adherent to it, so that it resembles a coagulum around a projecting substance, an excrescence, or a thread. We call this clot a *blood-plug*, a *thrombus*, and hence designate the state in which it occurs *plug-formation* or *thrombosis*. This change seldom happens in the capillaries. We must look for it in the heart, arteries, and veins, and especially in the second-sized vessels of the two latter. Schultz found that blood which was collected in fresh animal membranes, a portion of the intestines, for example, coagulated more slowly than that in the proper coats of the veins; an experiment which is easily verified by tying the vessels of a living animal. But it is not so clear that the formation of a clot is a sign of the death of the blood, but rather as showing the reciprocal relation of the parts by which the normal state of the blood is longer preserved.

As regards the composition of a thrombus, it may be described as differing from a simple blood coagulum by its distinctly stratiform construction, by its larger per centage of fibrine, and by its containing a greater number of colorless blood globules.

*Thrombosis begins either on the coat of the vessel, or in the entire mass of the blood contained in a portion of the vessel.* The first is the most common, and hence the complete occlusion of the lumen of the vessel occurs in the smaller number of thrombi. In the heart it is the invariable state; and in the arteries and veins it is the most frequent case. Besides a normal surface, if there be others, (an unusual thing,) as, for instance, a foreign body thrust into the vessel; it is as if a needle or a thread were carried through it; and so are here the abnormal surfaces which retain the first deposit of the clot. In the first of these, from often a very minute beginning, the blood-clot enlarges itself in a laminated fashion, so that it is seen sometimes more globular, sometimes cylindrical, unequal, rough, or villous in form. But the surface of the clots is always smooth, when particular influences are not operative, or when a first deposit is not made through the entire special unevenness of the surface of the vessel. The coagulated fibrine acts here by attraction on the fluid fibrine, in the same manner as we can hasten the coagulation of blood in the veins by the addition of a clot to that in which the coagulating process has not yet begun. (*John Davy, Schroder, Van der Kolk.*) The thrombus gradually grows in this way, so that by projections from the sides of the vessel the lumen of the latter is diminished and an entire obstruction ensues.

An obstructing thrombus naturally takes the form of the vessels in which it is impacted, and according as it is the normal or the reverse state, hollow or cavernous, will the plug be cylindrical, rounded, or branched. Its end toward the heart has a conical roundness, as well in the arteries as in the veins. In this way the plug always enlarges itself. Very commonly it is like what we are accustomed to find in ligature of an artery; that is, it extends from the point of obstruction to the next large collateral vessel. The thrombus itself is not so large at the lower part of the artery as it is at the next collateral branch. In the veins the circumstances are still more various. It shapes itself in obstruction of the right iliac vein as a long plug, commonly extending itself into the cava; but only seldom so in the left iliac, while the situation of the aorta favors a union of the mouths of the vessels. So after a single ligature of a vein at the upper part of the ligated vessel, in common only a smaller thrombus arises, reaching to the next valve; while Virchow obtained an extraordinarily long plug, extending as far as the heart, when he introduced a piece of caoutchouc into the vein and tied this so as to prevent a collapse of the vessels. Very generally it happens that the thrombus, especially of the veins and auricles, after it has more or less filled the lumen at its points of formation, continues beyond their limits, and particularly in the direction of the circulation, so that it gradually grows in a part of the vessels which hitherto had remained free and unobstructed. Large polypoid continuations are thus formed, which extend from the auricular processes into the auricles, and even through the atrioventicular valves into the ventricles. From the ramifications of the veins are formed bulb-like or cylindrical prominences into the main trunk. The author therefore distinguishes—1. The coagula originating on the side of the narrowed vessels; 2. The locally obstructing ones; and 3. The continued or permanent plugs.

After having shown the formation of a thrombus, Virchow next describes the conditions under which it is brought about. These are essentially reducible to two, namely, *congestion of the blood, and the altered molecular attraction between the blood and the surface of the vessels of the part.* He had in a preceding chapter spoken in detail of passive congestion, (*infarction.*) It may be regarded as a slow and interrupted movement of the blood, with, for the most part, a local increase in the quantity of this fluid. A continuance of this state tends more and more to a stasis, a stagnation (*stillstand*) of the blood, and lays the foundation for the coagulation of the fibrinous portion. We can at any spot give rise to its coagulation by an artificial interruption to the course of the blood, even when this fluid is in its natural state, if the interruption is not too suddenly removed. It appears that but a short time is required for the production of a clot in the interior of a vessel.

This obstruction of the circulating blood has been known for a long time, and the doctrines of the older writers on the corruption of the blood, on the conversion of the infarcted fluid into polypi, and finally into acrid, putrid, mucous, atrabilious matter, plainly grew out of such knowledge. The occurrence of thrombosis in ligated vessels; the coagulation in an aneurismal sac and in varices, were surely proofs, showing, beyond doubt, that the stasis or stagnation of the blood sufficed for the production of a coagulum. But the doctrine of the inflammation of the inner coat of the blood-vessels turned attention in an entirely different direction; and without inquiring seriously into the nature of the clot, or solving the question, whether the coagulated blood in an inflamed vessel cannot be accounted as a mechanical obstruction, the new idea of the flow of the exudation on the internal surface of the heart and arteries was adopted.

Virchow alludes to the experiments of Sasse, Bouillaud, Rigot, and Trousseau, Gendrin, and Corneliani, and mentions his having himself performed others of a new and extended series, which, as relates to the veins, have been repeated by Meinel, and to the arteries by Notta. It appears from them that by no kind of irritation or inflammation of the blood-vessels is there caused a coagulable exudation from the inner surface entering into their lumen; and that, for the most part, every exudation from the internal coat remains, and that the internal coat itself may be changed in its nutritive composition. No fluid exudation could be detected in the carefully conducted experiments of putting a double ligature on the vessels. But it seems that, not unfrequently, the internal coat is destroyed, and thus the way is opened for an exudation on the surface, (*Corneliani, Gluge, Luschka;*) so that, when an inflammatory irritation seizes the blood-vessel, in which blood yet flows, a clot of the latter can be formed so soon as the inner coat is penetrated, when also, for instance, through any means there is necrosis of the vessel. This clot acts precisely as globules of quicksilver or a needle in the heart, and as a thread, a piece of metal or of wood in the vessel, whether the surface be smooth or uneven; so that the form of the surface does not seem to be the cause of the formation of the clot, but the adhesion of the blood to an essentially changed structure of the surface.

Notwithstanding the compression exercised by Virchow in his summary account of the subject, we find that we must hereafter content ourselves with presenting the heads of the several propositions which he illustrates by reference to contemporary inquirers and to pathological facts. For these latter we must direct the reader to the original work.

Thrombosis, from passive congestion, may take place, as Virchow states, under the following circumstances:—1. *Obstruction or diminished diameter* (*narrowing of the lumen*) *of the vessels*, as after ligature of a vessel, or from compression of the veins by tumors, extravasation, enlarged uterus, induration of tissue of the liver, kidneys, and lungs, gangrene, tuberculosis, inflammatory hemorrhages. Obstruction in the capillary circulation may have the same effect as a ligature of an artery.

2. *Breach of continuity of the vessel.*—When a vessel is perforated or jagged through a pathological process, a thrombus ensues. From this cause we may have, after labor, an alarming traumatic or a puerperal thrombosis entirely independent of phlebitis.

3. *Dilatation of the vessel and of the heart* is one of the conditions for thrombosis, as in the familiar deposit of an aneurismal sac, the coagula of a varix, plugs in enlarged auricles and partial aneurism of the heart, the globular vegetations of Laennec between the dilated columnæ carneæ of the ventricles and the auricles; finally, the vermicular and nodulated coagula in dilated vascular plexuses, which are met with about the neck of the bladder and the prostate, and in the broad ligaments, and which go to the formation of *veinstones.*

4. *Absolute weakness of the heart's force.*—*Marasmus.*—Under this head we find most forms which have been spoken of collectively as spontaneous phlebitis. The credit is due to John Davy and Gulliver for being the first to show that a prostrated state of the powers of life and a languid or retarded circulation soon predispose to the formation of a coagulum. Hasse and Bouchut still more pointedly directed attention to cachexia, and states of chronic disease, besides a variety of other morbid changes, as productive of coagulation of the blood in the veins. This state of debility or of cachexia is denoted by the title of marasmus, in which specific reference to the nerves and blood is not called for.

It ought to be known that the chief ground for thrombosis is an enfeebled condition of the heart's power, which so much the more admits such changes when a local hinderance of another kind, such as obstructive congestion, or gravitation contributes. Virchow designates thrombosis occurring under these circumstances as a *marantic thrombosis*, which is an extremely frequent thing in severe fevers, as typhus, for example, and is a very common complication of other diseases,—pulmonary consumption, ulcerated cancer, chronic affections of the joints. In adopting the idea of phlebitis, it was not sufficiently attended to that the smaller veins are clear of disease, and that the obstruction exists in the larger trunks,—(crural, iliac, cava, jugular, the cerebral sinuses.) *In the majority of cases, the beginning of marantic thrombosis shows itself behind the valves of the veins, at the point where the valve unites with the coat of the vessel.* It appears, therefore, that in a state of marasmus, where in addition to debility of the muscular tissue of the heart, anœmia, and slight atony of the muscular coat of the vessel exist, the valves are frequently unable to meet, which will be intelligible through the feebleness of respiration, and partial obstruction of the vessels of the lungs, impeding the movement of the blood.

*Thrombosis from interrupted molecular attraction* is next entered on by Virchow.

1. Gangrene is the first specified under this head. Obstruction of the vessels through gangrene, artificially produced, will interrupt the course of the blood in the vessels both to and from the heart, and a narcotic agency will act directly on the blood. But a perforatory destruction of the coat of a vessel, as in ulcerative processes of the lungs, has, also, often caused a coagulation, and without there being an introduction into the blood of such substances by imbibition.

2. *Nutritive obstruction of the vessel, especially the inflammatory.*—This although, less common in the veins than is generally supposed, is still evident in different places. The author has seen, in the superficial veins whose coats were affected through metastatic abscess, especially adjoining purulent collections, a uniformly spread and laminated coagulum form over a circumscribed infiltration.

3. *Contact of the blood with foreign bodies in the coat of the vessel.* A thread introduced into a vessel, as was done by Magendie and Cruveilhier, and, as Virchow saw practiced on a dog, will give rise to complete obstruction of the lumen of the vessel. Some have enjoyed the opportunity of seeing a needle or other pointed body, which had perforated the heart, or a vessel, become lodged there: this was the beginning of a thrombosis. But there is another more singular state of things, viz. a *hemorrhagic thrombosis.*

Virchow states that a thrombus after its formation may be broken up and driven forward with the circulating blood to a remote part of the blood-vessel system where it becomes impacted. He claims to be the first to direct attention to this point in the arteries of the lungs, obstruction of which, through a blood-plug, had been made, a short time before, the subject of investigation by Dubini and Paget: he soon learned that there were analogous plug formations in the arteries of the body at large. Since then, he has published his first essays, (1846 and 1847,) on the transportation of plugs into the blood-vessel system,—a true metastasis. Confirmatory observations have been subsequently made by Doderkein, Ruhle, and Tuffnell, and also by Pioch, (1847,) Cruveilhier, (1849,) and Kirkes, (1852.) On the subject of priority of observation we would say, in the words of our English contemporary already named:—"To Virchow, however, the merit is due, not only of having explained that many cases of obstruction of the pulmonary arteries are the effects of the lodgment of fibrinous plugs carried there from a distance, and having pointed out the places where, and the circumstances under which, the primary coagula are formed; but to him, also, we must accord the priority in the question of the obturation of the systemic arteries, in consequence of the detachment of solid substances from the valves of the left ventricles, &c." (1845.)

As regards the effects of fibrinous clots on the inner surface of the heart and vessels, Virchow made experiments to test, by analogy, this question. He introduced into the veins of animals relatively large and hard bodies, such as caoutchouc, quicksilver, pieces of muscle, fibrinous clot, and a venous thrombus itself, and saw them carried into the circulation. The result was, as Dieffenbach had ascertained in the case of cholera, that the inner surface of the heart was unaffected by the pressure of these foreign bodies, as it was, also, by that of ice. The author draws the conclusion that the plugs formed in the venous blood, in the veins of the larger circulation, (the right heart,) may pass into the pulmonary arteries; and those formed in arterial blood, (pulmonary veins, left heart, and larger arteries,) enter into the arteries of the body, while, finally, those formed in the roots of vena porta, are carried into the ramifications of the vena porta hepatica, where they are locked up.

This would seem to be the opportune moment to make some mention of the observations of Mr. Kirkes in confirmation and elucidation of the position just laid down by Virchow. The paper by Mr. Kirkes* is contained in the *Medico-Chirurgical Transactions* for 1852. He assumes at the outset, as a fact clearly established and generally admitted, that the fibrinous principle of the blood may, under certain circumstances, separate from the circulating fluid during life, and be deposited within the vascular system, especially on the valves of the heart. "The parts of the vascular system within which these transmitted masses of fibrine may be found, will, of course, depend in a great measure, upon whether they proceeded from the right or the left side of the heart. Thus, if they have been detached from either the aortic or mitral valves, they will pass into the blood propelled by the left ventricle into the aorta and its subdivisions, and may be arrested in any of the systemic arteries or their ramifications in the various organs, especially those which, like the brain, spleen, and kidneys, receive large supplies of blood already from the left side of the heart.

"If, on the other hand, the fibrinous masses are derived from the pulmonary or tricuspid valves, the pulmonary artery and its subdivisions within the lungs will necessarily become the primary if not the exclusive seat of their subsequent deposition." A division being thus made, Mr. Kirkes records, first, the remote effects resulting from the separation of fibrinous or analogous deposits from the valves or interior of the left side of the heart; and, secondly, the corresponding effects produced by the detachment of like deposits from the valves or interior of the right side of the heart.

In the first three cases, death seemed to ensue from softening of the brain, consequent on obliteration of one of the main cerebral arteries by a mass of fibrinous material derived directly from warty growths on the left valves of the heart. Of the subjects, two were pale, delicate, and enfeebled females; the third was a man of intemperate habits, and, when admitted into the hospital, he was in a state of extreme emaciation and debility, had sloughs on his back and hemiplegia of the left side. In each of the three cases there was pale softening of the brain; a plug of fibrine obliterating the canal of one of the main cerebral arteries; masses of fibrinous deposit in the kidneys and spleen, and which seemed to be the source of the mischief elsewhere; large warty fibrinous excrescences in the left valves of the heart. Among other judicious observations on this subject, especially in its pathological bearings, Mr. Kirkes makes the following, in reference to partial and temporary paralysis suddenly ensuing in one or more limbs in young persons:—"This morbid condition may

* On some of the Principal Effects resulting from the Detachment of Fibrinous Deposits from the Interior of the Heart and their Mixture with the Circulating Blood.

be due," he thinks, "to the interruption of a due supply of nutriment to the brain by the temporary plugging up of a principal cerebral artery by fibrine detached from a diseased valve on the left side of the heart. Temporary loss of power in one or more limbs is not an uncommon circumstance in young persons affected with heart-disease."

We must not, by any means, suppose that the arterial branches at the base of the brain are "the only arteries in which fibrinous masses, detached from the valves of the left side of the heart and mingled with the stream, may be arrested. In Cases I. and II., coagula were found in the iliac and femoral arteries, and in Case III., in the renal; and in each of these cases coagula were, in all probability, derived from the same source as that which furnished the clot in the middle cerebral artery, namely, the warty masses on the mitral valve."

Mr. Kirkes next proceeds to offer a few observations on some of the effects which smaller portions of fibrine similarly detached, and arrested in minute arterial branches or in capillaries, appear capable of producing. One of these effects seems to be displayed by the singular masses of yellow, fibrinous-looking substance, not uncommonly found in the spleen, kidneys, and other organs, and hitherto described under such names as "capillary phlebitis," "metastasis," or "fibrinous deposits." But the more immediate object of Mr. Kirkes is "to show that these morbid appearances are very commonly associated with valvular disease of the heart, especially with those forms of disease attended with the deposition of fibrinous vegetations on the valves; and that, in the majority of cases, if not in all, they result from the direct transmission of particles of fibrine from the valves of the heart or elsewhere, and their subsequent arrest in the vessels of the parts in which these morbid deposits are found." The author has noted the coincidence of these deposits in the spleen or other parts supplied with blood directly from the left side of the heart, with disease of the valves or of the interior of the left side of the heart, in nineteen cases.

Mr. Kirkes next adverts to a third series of effects which sometimes result from the introduction of fibrinous particles into the circulating blood, namely, the manifestation of phenomena like those indicative of a morbid poison in the blood. A case is detailed illustrating this part of the subject. It was of a healthy-looking boy, but who had been lately badly off and much stinted for food: he was received into St. Bartholomew's Hospital with obscure typhoid symptoms and a petechial eruption on the skin. We have not room for the details of the disease or of the *post-mortem* appearances; but give the conclusions of the author, who says, "The pain on the right groin, with which the attack set in, was rheumatic; then ensued rheumatic inflammation of the mitral and aortic valves, with ulceration of the latter, and deposition of fibrinous deposits on both. From these fibrinous deposits, many of which were loose and easily detached after death, portions had probably separated during life; and, being mingled with the blood, were transmitted with it to all parts of the body; then arrested on the capillary net-works and smaller arteries, they produced the various petechial-looking spots on the skin and most of the serous and mucous surfaces of the body, the buff-colored blotches and streaks, and the solid, fibrinous masses in the kidneys and spleen, while they probably caused the fatal issue of the case by the pressure resulting from the extreme engorgement of the minute vessels of the pia mater and the substance of the brain." Taken in conjunction with others, this case seems to possess great pathological value, as illustrating the serious effects which may result from direct poisoning of the blood, by the products of rheumatic inflammation of the valves of the heart being mingled with it. In the typhoid symptoms noticed during life, and the secondary deposits found after death, the case presents features almost exactly parallel with those ensuing on phlebitis after wounds.

Mr. Kirkes concludes his valuable paper by a notice of the effects which may result from fibrinous deposits from the right valves of the heart. Here the lungs bear the brunt of the secondary mischief, displaying it in coagula in the pulmonary arteries, and various forms of deposit in the pulmonary tissue. We shall probably revert to this subject when we speak of Mr. Paget's paper on the formation of coagula in the pulmonary artery.

We fall back on Virchow, to exhibit his views of the mode of formation of *Embolus*. In contrast with that formed at its point of origin, the primary thrombus, which is made from a coagulum of blood, he designates the clot which has emigrated into another vessel, or more especially a wedged plug, as *embolus;* a distinction which is the more necessary, as it is not merely a thrombus (coagulum) which has been carried into distant vessels, but also other bodies, as, for instance, portions of the degenerated valves of the heart; and, as the author has elsewhere shown more in detail, the wedged plug serves as a point of accretion for new coagula on its surface, so that we generally find an embolus in the interior of a much larger thrombus. The location of the embolus is determined by the size of the detached bodies in relation to the diameter of the vessel. Larger bodies are sooner arrested and impacted in larger branches of vessels; smaller bodies, subsequently, in minuter ramifications. Without being able to say to what degree of minuteness in the size of the vessel these conditions can apply, we know that these bodies may still be contained in the extreme ramifications of the arteries. If, therefore, a solid body has once entered the capillaries, there can be no doubt of its capability of passing them on, so that we must regard the embolus as a leading arterial phenomenon. In general, the embolus is found at the point of bifurcation of an artery, or where the latter, by its numerous ramifications, is quickly diminished in size; the larger plugs generally rest astride, as it were, on the projecting points of bifurcation, while they are, also, in a measure, forced into the descending branches.

The emboli cause, as has been before shown, a partial obliteration (obturation) of the vessel, so that both before and behind them the vessel is open; and in the beginning they lie free in the lumen of the vessel. In both these contingencies they can be less distinguished from the original thrombus, especially when by their loose position, and by the perfectly unaltered state of the wall of the vessel, they indicate a greater duration. This was the reason why Virchow could not accede to the opinion of Baron, Cruveilhier, Paget, and Dubini, on the primary character of most of the plugs in the lungs, and why he here lays an especial weight on the distinction between the general primary obstructing, and the secondary or partial obstructing and emigrating thrombus. The next effect of the embolus is only an extreme diminution of the lumen of the vessel, not its entire obstruction; as few bodies are either in shape or in consistence calculated to adapt themselves entirely to the peculiar lumen of the vessel. It is, therefore, possible that blood can still pass between them and the coat of the vessel; the current of which, according to circumstances, is either increased or diminished,—yet so that, behind the embolus, there is always less blood admitted, and, therefore, there always occurs a stagnation. Very soon new deposits of coagulated blood take place on the circumference of the embolus, as well in consequence of the stagnation as of altered molecular attraction of the blood; and it may occur *that the embolus is entirely enveloped in the thrombus*, still without, on this account, obstructing entirely the lumen of the vessel. When Virchow introduced into the jugular veins of dogs triangular, prismatic pieces of caoutchouc, he found them, after some time, to be inclosed in a coagulated capsule in the pulmonary arteries, yet in such a way that there remained within the lumen of the vessel three small lateral canals.

If we suppose the embolus to be soft, it may be pressed against the walls of the vessel by the *vis a tergo;* or if it be very large, or cylindrical, the interruption of the lumen may be perfect, and an obturation be momentarily caused at the same time. Then that part of the artery situated behind the embolus contracts, if there is sufficient tonicity, and discharges the greater part of its contained blood. Before the embolus a secondary thrombus is formed, as in the application of a ligature, mostly up to the next large collateral vessels. Further details would lead us to a notice of the changes and metamorphoses of thrombus, which are sometimes retrogressive—(*softening, induration, petrifaction, and fatty degeneration;*) and sometimes of a progressive nature—(*organization, formation of areolar tissue, etc.*) In cases of organization, (John Hunter and Zwicky,) on the points of previously entire obstruction, the circulation may be, though imperfectly, re-established. Sometimes a lateral opening of the canal is established, that is, the thrombus adhering closely to one side, while the other part retracts and allows of the circulation. At other times there are formed irregular, or, at least, interrupted adhesions in the course of the vessel and canaliculization in the shape of sinuous degenerations. (Rokitansky.) Finally, we cannot doubt the formation of new vessels (vascularization) in the thrombus. (Stilling.)

Generally there occurs, at the circumference of the thrombus and embolus, a nutritive alteration in the wall which did not previously exist; and this change readily takes on an inflammatory character, as phlebitis and arteritis. The degree of change in the thrombus and embolus is dependent on the chemical and mechanical condition of the plugs, or of the parts from which they were originally formed. Under certain circumstances, as in a septic infection of the body, there may have been added to the original thrombus a certain amount of catalytic substance, which determines those bodies to assume a retrogressive character: in traumatic or puerperal thrombosis the intra-vascular coagula can receive, through contact with the septic surfaces, the germ of internal disintegration. If the embolus originates from a partially ossified valve of the heart, the mechanical irritation of the hard and irregular surface acts very energetically on the wall of the vessel at the point of obstruction. According to the degree of irritation there will be hypertrophy of the vessel, its adhesion to adjoining parts, infiltration of matter, formation of abscesses, and perforation of the wall of the vessel. If the irritation be of a chemical kind, there is a corresponding transformation of neighboring tissues.

*Thrombosis of the veins* is often so slight as not to excite attention; running its course without any consequences except inflammatory irritation of the walls of the vessels, as in hemorrhoidal tumors and varicose veins. Even in entire obstruction, and in thrombosis of the deeper seated veins, relief is afforded by free anastomosis of the contiguous vessels. Congestion and dilatation of the vessels dependent on this condition, may ultimately give rise to a cyanotic state, and thus enlarge our diagnosis. If the collateral circulation is insufficient, which is especially the case when this class of vessels is itself obstructed, (Cruveilhier,) there sometimes ensues *phlegmasia alba dolens*, or *ascites.* Through a gradual increase of the existing congestion are developed the higher degrees of venous hyperæmia, (*cyanosis,*) which very often rapidly disappears under the exosmosis of serous fluid from the vessels. This phlegmasia dolens in puerperal subjects, arising from an obstruction of the iliac veins, may also be met with in non-puerperal states, such as in phthisical subjects, cancer, and cachexia in general; and lastly it has been received as a general phenomenon.

Serous exudations generally occur in the neighborhood of the capillaries and the roots of the veins, not on the vein itself; and it may therefore happen that the point of exudation is far remote from the thrombus. When the collateral circulation is not re-established, and external irritations are conjoined, the inflammatory irritation may reach that point called *œdema calidum,* and then occurs generally a diffused erysipelas, which terminates in bullæ and purulent infiltrations, (diffused phlegmonous inflammations—pseudo erysipelas,) with diseased states of the neighboring lymphatic glands, and even with gangrenous destruction of the parts, in the shape of white gangrene. These states are most frequently met with in the phlegmasiæ of lying-in women. In some epidemics of erysipelatous puerperal fever we observe, from the beginning, a great tendency to the complication of local erysipelas with thrombosis and dropsy of the parts. Virchow's own experience coincides fully with that of Emmert and Cruveilhier, viz. that gangrene never occurs as a direct consequence of venous obstruction. He has observed cases of thrombosis of the lower extremities, extending to the vena cava inferior, not admitting the idea of collateral circulation, and in which there was nothing anomalous to be observed, except the œdema. He points out some degree of connection between these œdematous obstructions long continued and *elephantiasis.* Lastly, reference is made to the hemorrhages which occur in the highest degree of venous obstruction, especially in thrombosis of the cerebral sinuses, and hyperæmia and œdema of the pia mater. He has met with hemorrhagic infiltration of the subperitoneal and the deeper-seated subcutaneous tissues, also in the muscles and the surrounding areolar tissue, on the lower right side of the abdominal muscles, in a case of typhus in which there was thrombosis of the external iliac, hypogastric, and crural veins; whereas, a more limited thrombosis of the left common iliac vein only produced hemorrhage of the adventitious and subperitoneal tissues over a circumscribed space, in the posterior margin of the superior aperture of the pelvis.

*Thrombosis of the Arteries* is often a deuteropathic phenomenon, consisting of mortification and ulceration. Very frequently, from the mere fact of meeting with obstruction in the vessels leading to the gangrenous portion, as, for instance, in the lungs, it has been inferred that the obstruction caused the gangrene, but without foundation. The only important forms of thrombus and emboli are those which occur in consequence of the so-called atheromatous processes, (*arterio-sklerosa* of Lobstein.) The obstructions in the arteries, through primary thrombi, are distinguished from the obstruction caused by transported emboli, mainly by the suddenness with which the symptoms manifest themselves in the latter case, and the greater accompanying danger. With the emboli of the arteries on the brain are often associated *apoplectic phenomena*, and in those of the pulmonary artery appearances of *suffocation*. Sudden obturation of the coronary arteries would seem to cause acute paralysis of the heart, and the highest degree of the so-called *angina pectoris*, as may be proved by the application of a ligature on the coronary artery. The author speaks of the detachment of particles from any of the valves of the heart carried to the lower extremities, which is productive of obstruction, increasing sometimes to a great extent.

*The next effect of arterial obstruction is ischæmia in union with collateral fluxion.* Anæmia, coldness or algor, and diminished volume or collapse, become so great that the parts assume a death-like or cadaverous appearance, the more observable as this state is generally combined with a notable diminution of activity of the functions. The effects of ischæmia of the brain, after tying of the common carotid, have long been known; among these are noticed hemiplegia with or without convulsions, soporose and comatose states, and even death. From obturation by embolic plugs in the branches of the carotid in the brain, a great variety of symptoms has been observed, when not only the completion and rapidity of the circulation, but also the locality of the obturation must be borne in mind. The most common location is at the fossa of Sylvius, and the consequences of the ischæmia are immediately extended to the motor centres, so that the attack on such an occasion is generally very acute, mostly with a prolonged loss of consciousness, in the shape of *apoplexy*.

*In obstruction of the arteries of the extremities*, the most important symptoms occur on the sensitive nerves. Sometimes the only and yet doubtful symptom is decided neuralgia, which, on account of its lacerating character, is generally designated as simply rheumatic, and which may continue after the vessel has been restored to its former condition. The author has seen a case of cerebral paralysis after obturation, of a year's standing, in which, while the extensors were affected, there was permanent contraction of the flexors. In obturation of the cerebral arteries the sensibility is not affected, while the paralysis is complete.

The relations of the so-called hemorrhagic infarction, as for instance, in the lungs, the spleen, the kidneys, to the obturations of the vessels are not clearly understood.

The highest degree of retrogressive development from vascular obstruction is direct necrosis. After senile gangrene had been traced to arteritis, it had become a dogma that all the forms of gangrene were owing to the interrupted admission of arterial blood. But the experiments of Virchow, in addition to those of Enmert, have proved that senile gangrene depended least on primary obstruction of the arteries. Yet spontaneous gangrene, not specially appearing in advanced life, but as a particular consequence of rheumatic affections, is generally brought on by emboli and endocardial vegetations. This variety is characterized by mummification. It is never dependent on obturation when the nutritive vessels of the part are not affected by the latter.

We may infer, from Virchow's experiments, that local affections following the obturation of a vessel depend much less on the plugging itself than on the chemical and mechanical nature of the plug. The interruption in the courae of the blood in a tolerably large branch of the pulmonary artery, even in one of the trunks supplying a lobe, does not seem to exert any appreciable effect on the parts to which the blood is distributed by this channel, nor on the other lobes of the lungs, nor the rest of the economy. Virchow's experiments further show the formation of a distinct collateral circulation through the bronchial and intercostal arteries after the obturation of the pulmonary artery. A very important experiment on which a large number of pieces of muscle were introduced, exhibits the occurrence of death with all the symptoms of asphyxia, the heart having been found in the diastatic state. The injection of air acts likewise by the impediment it originates to the circulation through the lungs.

The obstructions of the pulmonary artery furnish the most numerous examples of emboli. In seventy-six subjects, examined after death, the author found twenty-nine of emboli of the pulmonary artery and pulmonary veins; and yet we are not able to show their great influence on the general state of the circulation, although we should look for their more frequent occurrence in parts through which the whole circulation is obliged to pass than in the case of obstructions of the arteries of the extremities.

Among the symptoms of the compensating hyperæmia have been noticed different congestions of internal organs, such as of the head, lungs, palpitation, abnormal pulsation, &c.; but the nervous are so blended with the vascular that it is hardly possible to draw a line of distinction between them.

Virchow terminates the chapter, in the *Manual of Special Pathology and Therapeutics*, on plug formations and obstructions of the vessels, by a brief account of the treatment of these morbid states. But, before we avail ourselves of this fact of his labor, it cannot be deemed amiss if we should make some observations on the changes which the coagulated fibrine in the heart and vessels frequently undergoes when left to itself, to be acted on by the blood and the vital forces to which it is subjected. For this purpose we would direct the attention of our readers to an interesting paper, by Mr. Gulliver, "On the softening of fibrine," as found in the twenty-second volume of the *Medico-Chirurgical Transactions*, (1839.) Mr. Gulliver makes a remark of no small practical moment, viz. on the frequency of obstruction of the veins by coagula, and which will we trust be not without its effect in quickening pathological inquirers, in this country, to a more careful and minute search after similar appearances, in their *post-mortem* examinations. Mr. Gulliver tells us, "Although the occasional obstruction of the veins by sanguineous coagula, whether softened or not, has become well known of late years from the labors of the continental pathologists, and particularly in this country [England] from several reports of dissections in the magnificent work of Dr. Bright, as well as from the essays of Mr. Arnott, Dr. George Burrows, and Dr. Copeland, my observations lead me to believe that the affection is not only more frequent than is generally supposed, but more concerned in disease, a circumstance to which my attention was first directed by Dr. Davy. Those who have not been in the habit, when examining dead bodies, of accurately attending to this point, would be surprised at the numerous examples of this condition noted in the register of dissections made under his superintendence at Chatham. The clots were, in most instances, partially whitened and softened in the centre; they were found in the heart, and also adhering to the inner membrane of the aorta; in the veins of the lower limbs, as well as in the cava, portal and renal veins; in the pulmonary vessels, and in the sinuses of the dura mater. They were chiefly observed in subjects who, having long suffered from various chronic maladies, had great prostration of the vital powers and languor of the circulation during the last days of existence; a state which was also remarkable in the cases related in this paper, and which seems more especially favorable to the formation and softening of the clot." Our readers need not be told how entirely these views, including the frequency of the occurrence of obstructing coagula and the part which they so often perform in disease, as well as the condition in which they mostly show themselves, are borne out and confirmed and explained in the masterly summary of Virchow.

Mr. Gulliver's object, in his paper, is to correct the misconceptions of those who believe that the changes in the substance, and especially in the interior of fibrinous clots, are due to the conversion of a portion of them into pus. Fibrinous coagula or concretions do, indeed, when exposed to a blood-heat for forty-eight hours become thoroughly softened, and present the color and consistency of pus, but they are distinguishable from the latter by the size of the globules and other tests, including a more ready putrescency. Softening of the fibrine has been noticed, moreover, without, in some cases, appreciable suppuration in any part of the body. Mr. Gulliver further contends that the purulent-like fluid found in the fibrinous clots of the heart and arteries is essentially distinct from pus, and analogous to, if not identical with, softened fibrine. And also, that the softening of coagulated fibrine is an elementary pathological condition of frequent occurrence, distinct from suppuration, and constituting a considerable proportion of the cases generally denominated suppurative phlebitis. Mr. Gulliver does not, however, deny, but, on the contrary, he has seen frequently the mixture of pus in considerable quantities with the pulpy fibrinous matter; "and, in two instances, some purulent globules were observed, completely insulated in the centre of a clot from the neighboring tissues. But, in both these cases, pus was also detected in the blood; and, when it is considered how frequently this contamination exists, it will not appear surprising that the particles of pus should become entangled in coagula contained in the vessels."

Reference has been made, more than once in our preceding remarks, to the paper of Mr. Paget, "On Obstruction of the Branches of the Pulmonary Artery," (1844,) in the twenty-seventh volume of the *Medico-Chirurgical Transactions*. The author makes an anatomical remark to be remembered, when we speak of pulmonary obstructions, whether congestive or inflammatory:—"From the arrangement of the pulmonary arteries, between which there is no anastamosis, except in their capillaries and smallest branches, it results that whenever the flow of blood through the capillaries of any part of the lung is prevented, there must also be a stagnation of the blood in all the branches from which those capillaries are derived; and in these circumstances the blood coagulates in the vessels and passes through various changes." These conditions are present in several diseases, such as pulmonary apoplexy, the advanced stages of pneumonia, arrested medullary matter in the lungs, which had become softened and entered the blood, and now, together with coagulated blood or fibrine, fills up nearly the branches of the pulmonary artery. The branches of this artery are often blocked up, also, by clots formed during life in those who die with great œdema of the lungs. In some of these cases the coagulation of the blood in the pulmonary artery may be regarded as a secondary phenomenon. But there are other classes of cases in which the formation of similar coagula appears to be a primary disease, or in which, at least, it cannot be regarded as the result of mere obstruction in the capillaries. Independently of other proofs to this effect, stress may be laid on the evident age of the coagula and certain organic changes which they have undergone. In one of the cases, given by Mr. Paget, he mentions its being distinguished by its laminated structure, as if formed by the deposition of successive layers from the wall toward the axis of the artery. This, it will be remembered, is described by Virchow as one of the chief characteristics of a thrombus.

In conclusion, we will now repeat what Virchow has to say respecting the *treatment* of obstruction and obturation of the vessels. This is confined to very narrow limits. We have no means for procuring the restoration of parts, a breaking up and removal of the plug. Surgery has been invoked as yet neither for thrombus nor embolus, and, as a general thing, it could hardly be ventured on. It is only in the various forms of gangrene of the extremities that amputation has been resorted to, with a view of limiting the gangrene, although, as yet, with unfavorable results, for we must wait until the operation can be undertaken with a clear insight into the nature of the circumstances. Chemical discutients, which were supposed to act by favoring the absorption of the fibrine, (nitre, carbonated alkalies,) have, in late times, lost their credit.

The first, best, and most natural termination is an areolar metamorphosis of the thrombus, which, as we have seen in a preceding paragraph, creates a partial restoration of the old canal. Next to this is a seasonable and sufficient return of the collateral circulation. As regards the first indication, it appears that the condition of the wall of the vessel mostly determines the kind of metamorphosis of the thrombus. The more irritated the wall of the vessel, or the greater its subjection to inflammatory irritations, especially if it is altered by putrid or gangrenous processes, the less disposed is the thrombus to a continued organization. We have next to take into consideration the state of the contiguous parts, especially where the vessel has been injured and the thrombus is in immediate contact with secreted and excreted fluids, which reach it by imbibition. Here it is necessary to preserve the utmost cleanliness of the parts, by means of topical bathing and injections; and to maintain the normal secretions by sometimes cooling, sometimes sedative, and sometimes stimulating treatment; to neutralize the septic agencies by chlorinated water, solutions of chloride of lime, diluted solutions of alum, acetate of zinc, and the tannates. In regard to the inflammatory condition of the wall of the vessel, this demands an antiphlogistic treatment, as has been often recommended since the time of Dupuytren. In such circumstances, local abstractions of blood are especially useful in the neighborhood of the obstructed vessel; also, applications of ice or of cold water, and inunctions with mercurial ointment. Conjointly with this treatment, the greatest possible quiet is to be observed, as motion not only interferes with the process of organization, but also causes destruction of the thrombus, the danger of which we have shown in speaking of venous plugs. In embolus of the extremities, the flexed position is the best borne, and favors the transverse dilatation of the vessels above the point of obturation. The regulation of the collateral circulation is the most difficult problem to be solved. There is generally, in the beginning, a collateral fluxion which is opposed by the state of the vessels, and may show itself in this way as active hyperæmia. If this is attended with immediate danger, on account of the magnitude of the difficulty, as is the case in great obstructions of the pulmonary arteries, we are obliged to resort to copious venesections, and to the use of dry and scarifying cups. These latter are especially indicated in cases of obstruction and obturation, which are often developed in anæmic and cachectic states of the system, where loss of blood would be very injurious. In moderate degrees of disease, and in parts reached externally, local depletion by means of leeches and cups will suffice; as, for instance, to the head and nucha, in obstructions of the vessels of the brain and of those of the extremities. These remedies have the advantage of allowing of repetition, and at the same time they prevent venous afflux, which may prove dangerous and favor gangrene.

It seems that cold, by its tonic action on the muscular coat of the vessels, is especially beneficial; its good effects are shown in neuralgia dependent on obturation. Reference is made to a case of obturation of the brachial artery, high up in the arm, in a young woman in the Hospital of Munich, which came under the author's observation, and in which intolerable pain of the hand was mitigated by applications of ice. He cites, also, the case of a patient in the Berlin Charity Hospital, in which there was obstruction of the crural artery, and in which the warm bath aggravated the pain: even touching the warm water produced a pricking sensation, particularly in the great toe, which lasted the whole day. In obturation of the arteries of the brain, cold is very useful for preventing collateral phenomena; especially, in this connection, may mention be made of a case of severe vertigo removed under its use.

It appears that, for a favorable issue of the case, it is better to moderate than to excite the collateral circulation. The general method of treatment, the object of which is to retain the heat in the parts deprived of blood, will consist of cataplasms and fomentations, and an increase of the activity of the circulation by aromatic and other exciting substances; but which have never exhibited satisfactory results unless in the latter stages of the disease. All irritations, which in themselves aid the dilatation of the vessels, fluxionary hyperæmia, lead to obstructions and contribute to the development of gangrene. Confounding spontaneous, or the embolic, with true senile gangrene, has been productive of much mischief. All the remedies which relax the walls of the vessels, and by this means aid in restoring the collateral circulation, act first upon the surface, where the want is not so immediately felt; and they have a very limited effect on the deeper-seated parts in which the more important anastomoses are situated. It is, therefore, better to waive this treatment, and to adopt the antiphlogistic course, at least, at the commencement.

In venous thrombosis, it is often possible to oppose, by local and general remedies, the obstruction of the circulation, or, at least, to diminish it. The best, and, at the same time, very important remedies, are the dietetic, —good, wholesome, easily digested food, slightly stimulating beverages to aid digestion, and often change of place. It is very important to watch the convalescent or the serious chronic cases in this particular. The motive power of the heart and the activity of respiration must be kept up to a certain degree of vigor, if we wish to prevent the plug-formation. But it is necessary, besides, to aid locally by an elevated position of the parts and graduated pressure by bandaging, so as to resist the determination of the blood; then resort to friction, removal of partial compression, etc.

In the traumatic forms of the disease we must endeavor, at the very commencement, to prevent the formation of large thrombi in the veins. It is, therefore, of the utmost importance, in the cases of puerperal patients, especially in the period of epidemic visitations, that the uterus should contract early, and with this view we have recourse to frictions of the abdomen with the hand, the external application of cold, and ergot internally. In operations, we must omit tying the veins, as otherwise we should prevent the discharge of blood from the lower portion of the vein next the wound; and hence it is useful, in venesection, to remove large coagula from the opening of the vein, as they may be increased so as to encroach on the lumen of the vessel.

If there be coagulations in the vascular system, efforts must be made to prevent their spreading, and especially their degenerence into emboli. To attain this object it is desirable to diminish the *vis a tergo;* but as in venous obstructions this would not so directly answer our purpose, we must endeavor to gain our end by local applications, and, especially, bandages, and at the same time enjoin the greatest possible quiet. The extremities are best supported by a sling.

Different from the cases hitherto considered are coagulations of the arterial blood, especially in the valves. Here the *vis a tergo* must be diminished, the contractile power relaxed, and the circulation brought down. Here, also, is a mild, cooling, and antiphlogistic treatment most appropriate; as by the administration of digitalis, nitrate of potash, cream of tartar, *aqua lauro-cerasus*, or hydrocyanic acid, acids in general, and cooling laxatives. If we are convinced that we can act antiphlogistically by the use of the neutral salts, this treatment should have the preference.

Finally, there occurs a series of symptomatic phenomena, mostly in arterial obstructions and obturations. To these belong more particularly those of a nervous nature, which often require special consideration. In cases of great pain it is necessary to have recourse to therapeutical means, as its continuance weakens the patient and interferes with his sleep. If local antiphlogistic remedies are insufficient, we have recourse to narcotics, such as opium, lactucarium, and, under certain circumstances, chloroform. As relates to this last-mentioned article, we consider ourselves justified in demurring to the recommendation of its use by the author, except in very rare and extreme cases, and in which the disease does not arise from obstructions in the heart or the pulmonary circulation. Fevers and gastric derangements open out special indications. The frequent occurrence of debility has given vogue to the tonic and stimulating treatment, by the use of bark, mineral acids, and wine; beyond, in fact, what it deserves in the early stage of the disease. In fever, from the absorption of septic substances, the treatment will vary with the circumstances of the case.

Our readers have now opened to them a wide, and, for the most part, a new field for pathological inquiry, and for novel applications of therapeutical agents. We may expect, in consequence, to find our pages enriched hereafter by many interesting details, from correspondents, bearing on the several points with which we have endeavored to make them acquainted.*

We must reserve for a future occasion a view of the connection between the theme just discussed and rheumatism, as set forth by Virchow.

* A laudable attempt, in this line, has been made already by Dr. Marcus Dana, in the Medical Examiner for 1850, entitled "Observations on Endo-Cardial Coagula." Dr. Meigs, in his Observations, to which reference was made in a preceding page, has chiefly directed attention to the occurrence of heart-clots after hemorrhages in females, during their puerperal state.

